# Application of machine learning in understanding plant virus pathogenesis: trends and perspectives on emergence, diagnosis, host-virus interplay and management

**DOI:** 10.1186/s12985-022-01767-5

**Published:** 2022-03-09

**Authors:** Dibyendu Ghosh, Srija Chakraborty, Hariprasad Kodamana, Supriya Chakraborty

**Affiliations:** 1 Molecular Virology Laboratory, School of Life Sciences, Jawaharlal Nehru University, New Delhi, 110067 India; 2 Department of Chemical Engineering, Indian Institute of Technology Delhi, New Delhi, 110016 India; 3 School of Artificial Intelligence, Indian Institute of Technology Delhi, New Delhi, 110016 India

**Keywords:** Machine learning, Deep learning, Plant virus, Pathogenesis, Host-virus interactions, Evolution and emergence, Control options

## Abstract

**Background:**

Inclusion of high throughput technologies in the field of biology has generated massive amounts of data in the recent years. Now, transforming these huge volumes of data into knowledge is the primary challenge in computational biology. The traditional methods of data analysis have failed to carry out the task. Hence, researchers are turning to machine learning based approaches for the analysis of high-dimensional big data. In machine learning, once a model is trained with a training dataset, it can be applied on a testing dataset which is independent. In current times, deep learning algorithms further promote the application of machine learning in several field of biology including plant virology.

**Main body:**

Plant viruses have emerged as one of the principal global threats to food security due to their devastating impact on crops and vegetables. The emergence of new viral strains and species help viruses to evade the concurrent preventive methods. According to a survey conducted in 2014, plant viruses are anticipated to cause a global yield loss of more than thirty billion USD per year. In order to design effective, durable and broad-spectrum management protocols, it is very important to understand the mechanistic details of viral pathogenesis. The application of machine learning enables precise diagnosis of plant viral diseases at an early stage. Furthermore, the development of several machine learning-guided bioinformatics platforms has primed plant virologists to understand the host-virus interplay better. In addition, machine learning has tremendous potential in deciphering the pattern of plant virus evolution and emergence as well as in developing viable control options.

**Conclusions:**

Considering a significant progress in the application of machine learning in understanding plant virology, this review highlights an introductory note on machine learning and comprehensively discusses the trends and prospects of machine learning in the diagnosis of viral diseases, understanding host-virus interplay and emergence of plant viruses.

## Background

### Machine learning: an introduction for biologists

Over the years, extensive research has been carried out in various fields of biology to understand the science behind a plethora of complex biological phenomena. The study of problems such as traits in plants and plant viral diseases lead to generation of massive data sets. The progress in technology has rendered data generation a simple task. Cost-effective technologies such as next generation sequencing (NGS) have made it easier to gather data regarding gene expression, chromosome conformation, genetic variation, traits and diseases of animals and plants, leading to generation of such massive data sets having multiple characteristics [1]. However, the resultant data explosion, especially in the field of omics, has made the handling of large datasets a major concern. The traditional statistical data analysis methodologies are not effective or efficient anymore in this context [2].

Furthermore, biological phenomena comprise various aspects, which lead to the generation of more than one data type. This necessitates an integrated analysis of the different types of data. But the noisiness of heterogeneous biological data makes this a difficult task [3]. Data dimensionality is another major impediment, for instance, omics data is generally highly resolved, hence highly dimensional. Moreover, the sample size in biological studies is limited in most cases. This may lead to issues including overfitting, multi-collinearity and data sparsity [4].

In order to overcome all these barriers, attempts are being made to incorporate machine learning (ML) and deep learning (DL) tools in the analysis of the datasets. ML tools identify patterns in the data using different statistical methods. Based on existing data, the ML paradigm can be used to derive models for classification, pattern recognition, and predictions. DL algorithms extract high-level features from huge datasets (such as collection of genomic sequences, or images), recognize the hidden patterns, and then utilize them to train models [5]. These trained models can be further applied to diverse types of unseen data from different sources for tasks such as prediction and classification. These techniques have the ability to tackle tough problems by detecting structure in seemingly random data, even when the amount of data is too complex and large for human comprehension [6]. Hence, ML especially DL, has the ability to perform analysis of enormous datasets in an extremely efficient, cost-effective, accurate and high-throughput manner [7].

In the context of ML, there are two primary frameworks for training the models: supervised and unsupervised learning. Both of these have potential for use in biology. Under supervised learning, the given collection features, or attributes of a system under investigation, are labeled [8]. Two recurring problems in the supervised learning framework are regression and classification. The classification process assigns objects into classes on the basis on the properties of features. In biology, one example of such training (involving mapping of object-to-class) is mapping of gene expression profiles to their respective diseases. The algorithm returns an assigned class of the object with certain “confidence measure” indicating the correctness of classification. Some of the widely used supervised models are linear/nonlinear regression, support vector machines (SVM), Gaussian processes, and neural nets [9].

In unsupervised learning, the objects involved in the study are not under any predefined labels [10]. The entire goal of these models is to recognize similarities in the various objects by exploring the data. These similarities define clusters in the data (groups of data objects). So, the basic concept of unsupervised learning is to discover natural patterns in data and categorize it into groups. Overall, in supervised learning, the data is pre-labeled and the algorithm learns how to utilize the labels to associate the objects to the classes. On the other hand, in unsupervised learning, the data is unlabeled, and the algorithm also learns to create labels by clustering the objects. Principal component analysis is an important example of unsupervised learning technique which includes k-means clustering, Gaussian mixture models, density-based spatial clustering of applications with noise (DBSCAN), and hierarchical clustering [9].

In certain exceptional cases, a method known as semi-supervised learning has proven to be quite useful. One example of such a scenario could be the classification of protein sequences. Only a few samples of protein sequences are labeled (belonging to a known class) but numerous sequences belong to unknown classes. The semi-supervised algorithm combines a small amount of labeled data with a large amount of unlabeled data during training [11].

**Fig. 1:**
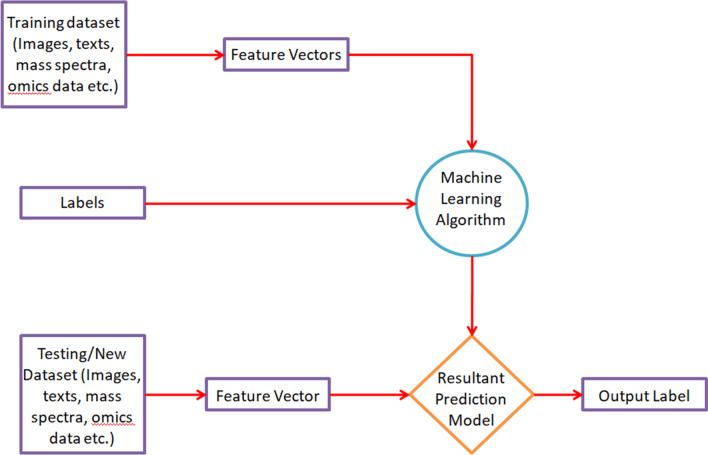
Standard flowchart for creation of a machine learning model to study biological data. The figure here shows the steps followed in order to create a machine learning model that can successfully study different types of biological data. The data is initially split up into training and testing sets. Each object of the training set is associated with a feature vector, which is passed into the required machine learning algorithm. After manipulating the various parameters of the model, a resultant machine learning model for prediction is developed. This model is then checked by passing the objects of the testing set through it. The resultant output accuracy determines the usefulness of the created model

The basic steps for creating a machine learning model for the study of biological data are shown in Fig. 1. Following the collection of data (labeled or unlabeled), it is divided into two sets for training and testing. The data samples need to undergo preprocessing and augmentation before the splitting in case they are corrupted with noise and outliers. Next the model is trained using the training dataset. The model can either be created from scratch, or a pre-trained model can be adjusted according to the collected dataset. Once the trained model is ready, the testing data is fed into it to determine the accuracy with which the objects are classified into different labels [12].

**Fig. 2:**
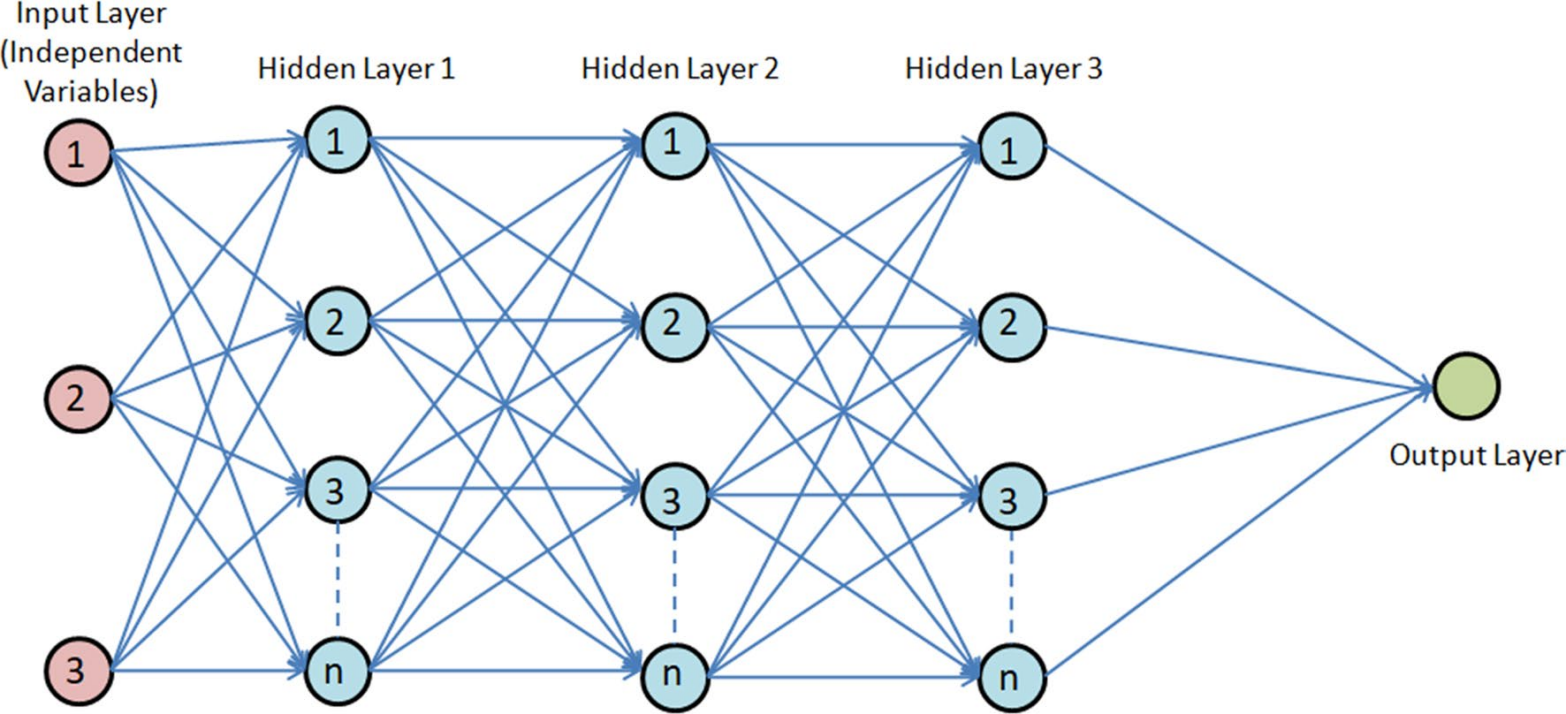
A schematic representation of a standard artificial neural network. The network is divided into three major components: the input layer, multiple hidden layers and the output layer. In this figure, it is assumed that the input layer has 3 independent variables, each of which is parsed through a set of weights and activation functions in the hidden layers and finally output layers to yield the model output. The activation functions are nonlinear mathematical function such as Tanh, Sigmoid, ReLU, etc. to induce nonlinearity to the model. Depending on the network structure, there may be ‘n’ neurons (also called hidden layer units) in each hidden layer and there may be multiple hidden layers. Any ANN with more than one hidden layer is technically is deep ANN. Once an input is fed into the network, one after another, each hidden layers gets operated among each other till finally the output layer is reached and activated, producing the final result. Weights in each layer is trained by means of the backpropagation algorithm

Deep learning is broad category of machine learning wherein large multiple layered neural network models are employed for representation learning. Deep learning can be performed in supervised, unsupervised, or semi-supervised fashion. When working with neural networks, we essentially attempt to create the inferences analogous to the human brain by building an artificial neural network (ANN) [13]. An ANN resembles a biological neural network. The artificial neurons used here are basically mathematical models that carry out three main functions: activation, addition and multiplication. The goal is to build layers of neurons, each of which produces a suitable response to any input provided to it. The neurons of each layer multiply their inputs with the corresponding weights. Then it is passed through the activation function and finally transferred to the next layer of neurons. Once the input layer is fired up, the decision moves along to the subsequent layers of the neurons (hidden layers), firing up the respective neurons until the final output layer is reached [14]. A schematic representation of a neural network is presented in Fig. 2, where the various components of the network such as input, hidden and output layers are explained.

In neural networks, the direction of information flow is determined by the internodal connections. On this basis, there are two classifications of neural networks: (i) unidirectional flow which can further be divided into cascade forward and feed-forward; and (ii) bidirectional flow, also known as recurrent flow [15]. In feed-forward networks, the flow of information between the layers takes place in one direction. Cascade forward is similar except that the input to the next layer is weighted. In recurrent networks, flow of information takes place in both directions. All the nodes are interconnected among each other, including self-connection. These networks are extremely complex, bulky, difficult to operate and take up a large amount of computational space. In addition to this, some neural networks architectures, such as self-organizing networks, convolutional neural networks (CNN), variational auto encoders (VAE) and generative adversarial networks (GAN) [16, 17] have recently attracted great attenttion in the DL community.

Various parameters are used to evaluate the classification performance of the developed model. Some of the important parameters include accuracy, sensitivity/recall rate, specificity rate, precision/positive predictive value, negative predictive value and F1_score_ [18]. The performance of models is evaluated by calculating various ratios involving true positives, false positives, true negatives and false negatives. All of these can also be combined into a single confusion matrix to assess the model’s performance [19]. Furthermore, the phenomena of overfitting and underfitting are widely faced while employing ML models [20]. Overfitting occurs when the model is fitted with respect to the noise in the data rather than the underlying signal. The validation data error increases while the training data error decreases [21]. On the other hand, underfitting is the reverse scenario. In this case, the model is not capable of recognizing data variability [22]. Several techniques including penalty methods, training by early stopping, batch normalization, and dropouts are being developed to avoid such imperfect fittings [23].

## Main text

### Application of machine learning in understanding plant viruses and viral infections

#### Diagnosis and detection of plant viral diseases

Plant viruses pose major economical constraints in cultivated crop plants across the world. Early detection of plant viral infection is crucial for successful disease management. An empirical evaluation through visible survey is traditionally followed by farmers to identify the symptoms of virus infected plants. The visual assessment bias dictates the inefficiency and inaccuracy of this method. On the other hand, laboratory-based detection techniques are primarily reliant on polymerase chain reaction (PCR) and serological-based method such as enzyme linked immune sorbent assay (ELISA). Despite their improved accuracy, the requirement for professional experts and their time-consuming and invasive nature pinpoints the shortcomings of these diagnosis assays [24, 25]. A pathogen attack significantly alters the biochemical and biophysical state of the plant leading to an alteration of tissue structure, water level and transpiration rate, ultrastructure of chloroplast and pigment content [26, 27]. At the very beginning of the twenty-first century, a few studies used remote sensors to capture and detect altered leaf reflectance and thermography profiling of diseased plants, which empowered the scientific community with an edge in phenotyping of stressed plants [28]. However, this technique was unable to determine if the stress was biotic or abiotic, and if biotic, what kind of pathogen was involved. Hyper-spectral imaging (HSI) and ML assisted data analysis are now revolutionizing the concept of stress phenotyping of diseased plants by enabling the diagnosis of specific plant diseases and even the severity of the disease. In the case of HSI, a light spectrum with a larger range of wavelengths is being used to capture plant images, which enables us to go beyond the limited range of human vision (400-700 nm) in monitoring minor alterations in the growth and development of plants [29]. For ML assisted detection of plant viral diseases, first a ML model has to be trained with a training dataset (images of diseased plants captured through unmanned aerial vehicle, grounded robots or even smartphones) [30–38]. There are increasing numbers of free online databases which provide images of specific plant diseases as training datasets. ‘Plantvillage’ is one such initiative [39]. Once a certain ML model has been trained accurately and precisely, a testing dataset (eg: hyperspectral images of specific plants under diagnosis) can be assessed [32, 34]. HSI generates high dimensional data with redundant information and hence, an efficient pre-processing of the data is crucial for the precise functioning of the model. An effective specific range of wavelength can be determined to reduce the dimensionality of HSI data [29]. The next step is feature extraction which minimizes the number of the features present in the raw dataset. The feature extraction method is vital for assuring a simple classifier with a limited variety of features, since multifeatured classification always hinders the smooth performance of the concerned model [40, 41]. ML researchers have devised a variety of feature extraction techniques based on the nature of the data and the model. However, the process is time-consuming and the success of the operation greatly relies on the expertise of the professional. Here comes the benefits of using DL techniques for feature extraction as DL empowers automatic extraction of features rather than handcrafted method used in traditional ML algorithms, for instance the application of convolutional neural networks. DL has substantially improved the reliability of plant stress phenotyping by enabling the accommodation of a large sample size for training and testing [40]. A major constraint of this method is the vast variation of environmental conditions between the field and the lab. While consistent temperature, humidity, and light intensity are maintained in the lab, all of these variables are constantly changing in the field, influencing the captured images [42]. Hence, it is recommended to use field images to train a model since it has been demonstrated that a classifier trained on field images can also classify lab-based images with precision [43]. The lack of availability of a huge collection of field-based images of a specific plant disease is another key challenge for an accurate and reliable diagnosis.

**Fig. 3:**
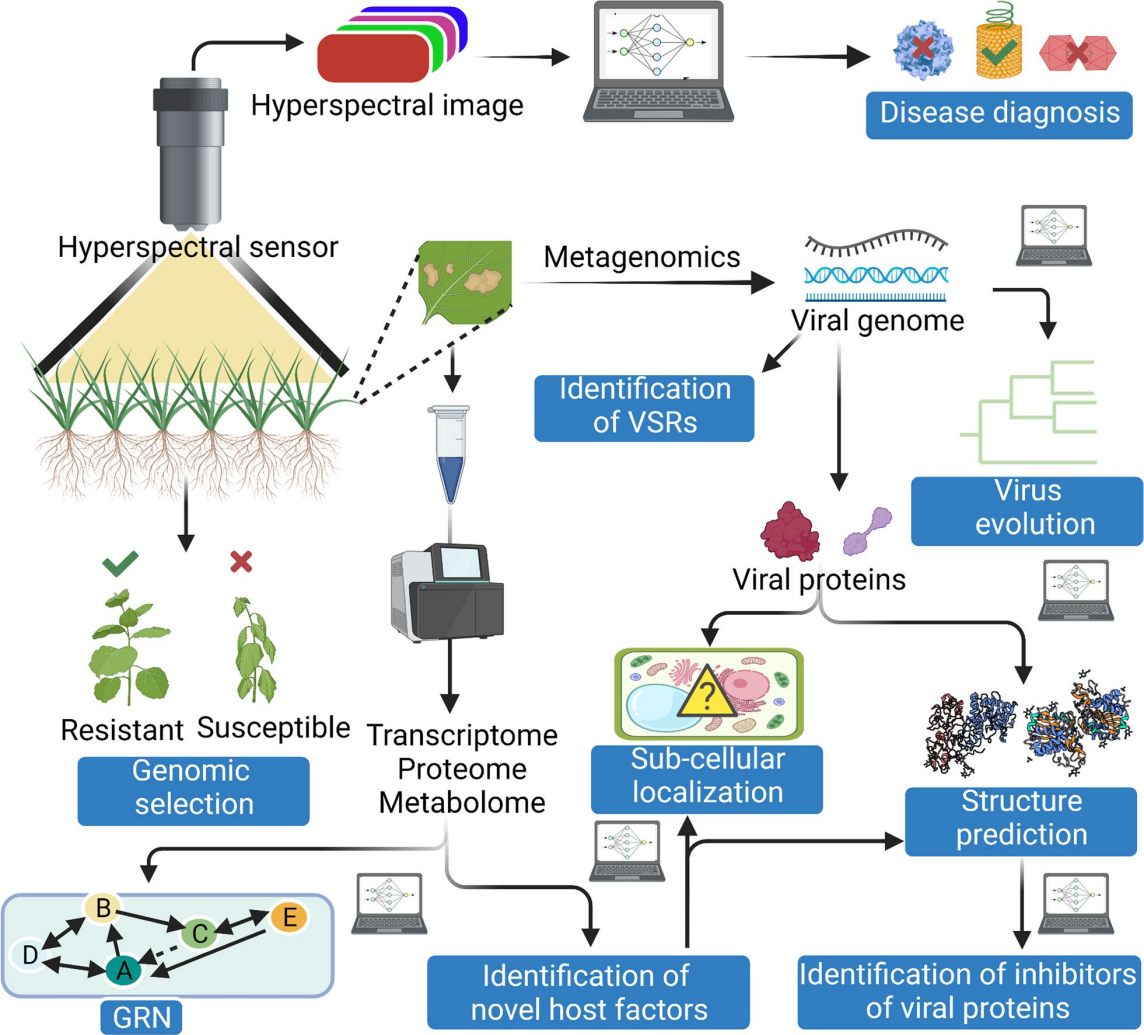
Application of ML in understanding plant virus pathogenesis. ML enables early diagnosis of plant viral diseases at field level through analyzing hyperspectral images. Metagenomics study of diseased plant samples helps identification of related and unrelated viral genomes. ML can assist in the classification of these viral sequences which primes our understanding of virus evolution. Furthermore, ML-assisted bioinformatics tools have been developed to identify viral suppressors of RNA silencing (VSRs). ML can also guide us to predict the sub-cellular localization and even the structure of the viral proteins. Prediction of accurate structures of virus encoded proteins may help to identify inhibitors of these effector proteins. To understand the host response, several groups have performed transcriptome, proteome and metabolome of virus infected plants. ML can prime the accurate and fast analysis of these high throughput data to identify gene regulatory networks (GRN) and novel host factors involved in host-virus interplay. Characterization of these host factors in terms of sub-cellular localization and structure prediction will boost understanding of plant virus pathogenesis. ML may also assist plant virologists in genomic selection to identify elite virus resistant cultivars. This figure was created using BioRender (https://biorender.com/)

Transfer learning is a recent advancement in the field of ML which enables the data scientists to adopt a previously well-trained model for solving a similar kind of problems [40]. For example-a model trained for chilli-leaf curl disease detection may be used for detecting leaf curl symptoms caused by viruses in tomato (Fig. 3). There are several approaches to adopt a pre-trained model; one can select and finetune the architecture and/or parameters of a model depending upon the types of datasets. Table 1 summarizes the development of ML assisted diagnosis of plant viral diseases over last few years.

**Table 1:** The application of ML-assisted diagnosis of plant viral diseases

Plant	Viruses/viral diseases	Algorithms/methodologies used	Accuracy	References
Cassava	Cassava mosaic disease	Convolutional neural networks (CNN)	96%	[31]
	Cassava brown streak disease		98%	
Cucumber	(i) Melon yellow spot virus; (ii) Zucchini yellow mosaic virus	CNN	94.9%	[30]
Mungbean	Yellow mosaic disease	CNN	91.234% for VirLeafNet-1	[37]
			96.429% for VirLeafNet-2	
			97.403% for VirLeafNet-3	
Potato	Potato virus Y	Support vector machine (SVM) classifier	89.8%	[36]
Sweet pepper	Tomato spotted wilt virus (TSWV)	Outlier removal auxiliary classifier generative adversarial nets (OR-AC-GAN)	96.25% (before the onset of visible symptoms)	[32]
Tobacco	Tobacco mosaic virus (TMV)	Successive projections algorithm (SPA) with extreme learning machine (ELM) classifier	98.33%	[34]
Tobacco	TMV	SVM	93.5% on the training set	[41]
			92.7% on the independent set	
Tobacco	TSWV	Model by boosted regression tree (BRT) algorithm and Wavelength selection by SPA	85.2%	[35]
Tobacco	Tomato leaf curl New Delhi virus and Tomato leaf curl Gujarat virus	CNN [Visual Geometry Group 16]	97.21%	[38]
Tomato	Groundnut bud necrosis virus (GBNV)	SVM	97.8%	[33]

#### Understanding the diversity and emergence of plant viruses

The recent trend of studying plant virome through metagenomics has unveiled the diversity of plant viruses. Huge numbers of phylogenetically related and unrelated virus species have been found in diseased samples [44, 45]. Explosion in virome data generated through NGS necessitates the urgent structural orientation and analysis of sequence data in order to understand the actual portrait of the viral diversity. Although a significant progress has been followed up in the case of animal viruses, limited efforts have yet been recorded in the field of plant virology [46, 47]. V-pipe has provided a bioinformatics pipeline for analyzing genomic diversity of human immunodeficiency virus (HIV) from sequencing data [48].

As RNA viruses use error-prone polymerases during their replication, the chances of mutations in their genome sequences remain quite high. Mutation in the viral genome finally leads to the emergence of new virulent viral strains [49]. A neural network-based model can predict probable point mutations in the RNA sequence. It has been successfully explored in the case of newcastle virus [50], and its optimized form may be very useful for the prediction of mutations in plant viral genome (Fig. 3). Besides RNA viruses, DNA viruses also possess significant genetic variations. Events like recombination and genome reassortment play crucial role in mediating the emergence of new viral forms [51, 52]. The identification of novel virus and satellite molecules through metagenomics approach emphasizes the importance of precise taxonomic classification followed by demarcation of these new species. An excellent effort by Silva and collaborators have developed Fangorn Forest, a ML based method, for classification of geminiviruses. Among the three tested algorithms, random forest (RF) has proven to be best in classification of genes and genera of this largest plant virus family [53]. Recently, a CNN guided sequencing platform has successfully completed human genome sequencing within couple of hours and efficiently identified the disease-causing variations in the genome. This ML based fastest sequencing approach may open up new windows in studying diversity and evolution of plant viruses [54].

#### Understanding host-virus interplay

Being obligate parasites, viruses rely on cellular machineries of plants for every aspect of pathogenesis including replication, gene expression and movement [55]. Plants elicit a robust antiviral immune response to restrict viral invasion [56]. Viruses encode effector proteins which disarm plant defense signaling. This tug of war continues which fuels the co-evolution of both virus and host [57]. Hence, understanding the interplay between plant and viruses is crucial for an in-depth dissection of viral pathogenesis.

Although plants have evolved a variety of tools and tactics to prevent virus multiplication, the resistance (R) protein-mediated immune response and gene silencing are the most well-known features of their antiviral defense [56]. A majority of canonical R-proteins contain nucleotide binding site leucine-rich repeats (NBS-LRR), which mediate direct or indirect recognition of virus-encoded effector proteins, resulting in the activation of effector triggered immunity (ETI). Very few *R*-genes imparting immune response against viruses have been identified and characterised till date, which limits our knowledge regarding the detailed mechanism of dominant resistance in plant virus interaction [58]. Support vector machine-assisted development of a high throughput bioinformatics tool, NBSPred, precisely identifies NBS-LRR containing R proteins from genome, transcriptome and proteome data [59]. Receptor-like kinases (RLK) are crucial players in the immune perception of phytopathogens, many of them acting as pattern recognition receptors (PRRs) which lead to induction of pattern triggered immunity (PTI) [60]. However, several plant viruses target RLKs to promote viral pathogenesis [61]. Brustolini et al. have recently developed a machine learning assisted technique for detection of RLKs from proteome data. Identification and annotation of novel RLKs may advance our current understanding of plant-virus interactions [62]. Furthermore, to identify host factors differentially regulated in host-virus interplay, several groups have performed transcriptome and proteome analysis in both resistant and susceptible plant varieties. These studies have revealed that a significant proportion of differentially expressed transcripts are of unknown nature suggesting the existence of novel gene regulatory networks (GRNs) modulating the host-virus interaction [63–66]. ML helps biologists to predict GRNs from high-throughput transcriptome data [67] which may lead to identification of several regulatory nodes of plant immune signalling (Fig. 3).

On the other hand, viruses encode few but multitasking effector proteins which facilitate the viral pathogenesis. Examining the sub cellular localization of these effector proteins is important to understand their mechanism of action. Furthermore, viruses also redirect the subcellular localization of several host proteins to disrupt their assigned functions [68]. ML assisted development of online tools such as LOCALIZER and MU-LOC enable precise as well as accurate analysis of subcellular localization of effector proteins and host factors by simply using amino acid sequences of proteins as input (Fig. 3) [69, 70]. Application of ML in the successful prediction of fungal effector proteins has added an extra edge in phytopathology research [71]. In the case of viruses, some viral effector proteins have been evolved to block antiviral gene silencing, known as viral suppressors of RNA silencing (VSRs). VSRs expand the negative impact of viral diseases by promoting synergistic associations among different plant viruses [72]. Jagga et al. have developed a bioinformatics platform, pVsupPred, for the prediction of VSRs encoded by plant-infecting viruses. They have used four classifier models including LibSVM, J48, Naı¨ve Bayes and RF, and among all of them, RF algorithm has emerged as the best with an overall accuracy of 86.11% [73]. Later on, in another study, sequential minimal optimization (SMO) algorithm had been improved to achieve an overall accuracy of 95.3% for the successful identification of plant virus encoded VSRs (Fig. 3) [74].

Another significant facet of plant-virus interaction is the virus induced alteration of microRNA (miRNA) homeostasis which impacts the transcriptome profile of the infected cells. Hence, it is important to identify the accurate targets of specific miRNAs regulating plant immunity and viral pathogenesis [75]. The advent of ML in advancing the scope of bioinformatics has significantly eased this difficult job. Supervised ML approaches including graphical models, kernel machines and evolutionary algorithms are being widely used to identify the specific miRNA targets in eukaryotes [76]. Further, a new category of DL models known as graph neural nets (GNN) is emerging as a promising tool in bioinformatics. The biological networks, based on small RNAs–disease associations, can be constructed as graphs with nodes and edges. GNN can operate on the graphical data and has more representative features, which can be efficiently used for inferences [77].

Finally, the best possible way to understand the functional aspect of a protein is to visualize its accurate structure. A very small proportion of plant proteins involved in immune signalling have been structurally characterised yet. In addition, structures of plant viral proteins are also largely unresolved. Labour intensive methods of protein crystallization is the major bottleneck here. However, Jumper and collaborators have revolutionised the idea of protein structure prediction by launching Alphafold2, a neural network-assisted structural bioinformatics platform, which can successfully solve a protein structure with almost equivalent experimental accuracy even if there is no similar protein structure available [78]. ML-guided docking studies efficiently screen chemical inhibitors of severe acute respiratory syndrome coronavirus 2 (SARS-CoV-2) encoded spike (S) protein [79]. Similarly, structure prediction of plant viral proteins and the prediction of their chemical inhibitors followed by successful delivery will be a novel and effective virus management strategy (Fig. 3).

## Conclusions and future perspectives

Plant-infecting viruses not only compromise the yield of the infected plants but also significantly affect the nutritional content of crops. Heavy crop losses due to plant viral outbreaks is a vital concern for global food security and hence necessitates the urgent implementation of smart management measures. Studies aiming to understand the evolutionary biology of plant viruses and the molecular biology of plant-virus interactions have generated large-sized datasets in recent years. Here comes the prospective role of ML. Although the last decade has witnessed a sharp increase of application of ML in solving complex biological problems [80], its usage in the field of plant virology is still at a very naïve state. Several reports highlighted the role of ML in the precise diagnosis of plant viral diseases [30–38]. Plant virologists can foresee the tremendous scope of ML in addressing virus evolution, emergence, plant-virus interplay and above all management strategies (Table 2). Moreover, several specific issues need to be explored.

**Table 2:** Brief description of ML-based bioinformatics platforms used in studying plant virus interactions

Name	Application	Input and output	Salient features	References
V-PIPE	Assess genetic diversity of viral population and ensure identification of true viral variants from high throughput data	**Input**: raw sequencing data (FASTQ format)	A hidden Markov model-based read aligner, ngshmmalign, is developed	[48]
		**Output**: viral diversity in terms of single nucleotide variants, local and global viral haplotypes		
NBSPred	Identify potential NBS-LRR and NBS-LRR like proteins	**Input**: Genome, transcripts and protein sequences	Gene prediction tool, Augustus2.7, is used to convert genomic sequences to protein sequences	[59]
		**Output**: Identification of NBS-LRR and NBS-LRR like proteins	TransDecoder is used to convert transcripts sequences to protein sequences	
			(i) Frequency of aminoacids, dipeptides, tripeptides and multiplet; (ii) charge (iii) hydrophobicity are considered for the calculation of sequence compositional property	
LOCALIZER	Predict the sub cellular localization of plant proteins and effector proteins encoded by plant-infecting fungus and oomycete	**Input**: sequence of plant proteins and eukaryotic effector proteins	Trained by support vector machine model	[69]
		**Output**: (i) probability of localization of a protein in nucleus, chloroplast or mitochondria	Maximum range: 2000 sequences	
		(ii) Identification of transit peptides (for chloroplast and mitochondria) and nuclear localization signal (NLS)		
MU-LOC	Predict the mitochondrial localization of plant proteins	**Input**: protein sequence (FASTA format)	The predictor has been trained using support vector machine and deep neural network	[70]
		**Output**: sub cellular localization		
pVsupPred	Predict RNA silencing suppressor activity of viral proteins (VSR)	**Input**: sequence of viral proteins	Random forest model guided tool	[73]
		**Output**: (i) prediction score, (ii) Whether positive VSR or negative VSR	Prediction on the basis of presence of (i) GW/WG motif and (ii) dsRNA binding domain in the viral protein	
Alphafold	Predict the structure of a protein	**Input**: amino acid sequence of a protein	Neural-network based model	[78]
		**Output**: 3D structure of the protein	Median accuracy: (i) 6.6 Å for Alphafold, (ii) 1.5 Å for Alphafold2	
Virfinder	Identify sequences of viruses from metagenomic data	**Input**: assembled metagenomic data	*k*-mer based prediction tool has been made using a trained logistic regression model	[81]
		**Output**: true viral contigs		

Firstly, a vast amount of OMICS data (including transcriptome, proteome and metabolome) of virus infected plants are available. The application of ML may enable the integration of these OMICS data which will definitely uplift our knowledge of host response, especially the impact of novel potential host factors in viral infection. Secondly, the coordinated application of ML and HSI pave a new path in the detection of viral diseases. Now, designing improved DL-based algorithms for easily accessible mobile app-mediated detection of plant viral diseases is the need of the hour. Thirdly, ML algorithms would be very helpful in deciphering the patterns and parameters of plant viral evolution. ML may enable the accurate prediction of recombination, rate of nucleotide substitutions, mutations and phylogenetic relatedness among viruses. The use of ML to predict the advent of aggressive recombinant plant virus strains and the likelihood of associated epidemics will be extremely beneficial. Fourthly, a significant improvement can be achieved in metagenomics data analysis through ML approaches. Along with viral reads, metagenomics data also contains a substantial proportion of host contig contaminations which often hinders the identification of small viral reads. VirFinder is one such *k*-mer based platform which enables the identification of prokaryotic virus sequences from mixed metagenomic data [81]. A similar approach can be employed for plant virome study. Fifthly, ML is now widely utilized in genomic selection for rapid and better prediction of superior genotypes for breeding purposes [82, 83]. A certain progress of ML assisted genomic prediction will definitely help breeders in developing elite virus tolerant/ resistant varieties. Finally, a collaborative effort by both plant virologists and big data analysts is of prime importance for the fruitful application of ML in the understanding of plant virus pathogenesis followed by the development of antiviral strategies.

## Data Availability

Not applicable.

## Abbreviations

NGS: Next generation sequencing
ML: Machine learning
DL: Deep learning
SVM: Support vector machines
DBSCAN: Density-based spatial clustering of applications with noise
ANN: Artificial neural network
CNN: Convolutional neural networks
VAE: Variational auto encoders
GAN: Generative adversarial networks
PCR: Polymerase chain reaction
ELISA: Enzyme-linked immune sorbent assay
HSI: Hyper-spectral imaging
HIV: Human immunodeficiency virus
RNA: Ribonucleic acid
DNA: Deoxyribonucleic acid
R protein: Resistance protein
NBS-LRR: Nucleotide binding site leucine-rich repeats
ETI: Effector triggered immunity
PTI: Pattern triggered immunity
RLK: Receptor-like kinases
PRRs: Pattern recognition receptors
GRNs: Gene regulatory networks
VSRs: Viral suppressors of RNA silencing
RF: Random forest
SMO: Sequential minimal optimization
miRNA: MicroRNA
GNN: Graph neural nets
SARS-CoV-2: Severe acute respiratory syndrome coronavirus 2
S protein: Spike protein
CNN: Convolutional neural networks
OR-AC-GAN: Outlier removal auxiliary classifier generative adversarial nets
SVM: Support vector machine
SPA: Successive projections algorithm
ELM: Extreme learning machine
VGG 16: Visual geometry group 16
BRT: Boosted regression tree
TSWV: Tomato spotted wilt virus
GBNV: Groundnut bud necrosis virus
TMV: Tobacco mosaic virus
